# Contralateral Regional Recurrence in Lateralized or Paramedian Early-Stage Oral Cancer Undergoing Sentinel Lymph Node Biopsy—Comparison to a Historic Elective Neck Dissection Cohort

**DOI:** 10.3389/fonc.2021.644306

**Published:** 2021-04-23

**Authors:** Rutger Mahieu, Inne J. den Toom, Koos Boeve, Daphne Lobeek, Elisabeth Bloemena, Maarten L. Donswijk, Bart de Keizer, W. Martin C. Klop, C. René Leemans, Stefan M. Willems, Robert P. Takes, Max J. H. Witjes, Remco de Bree

**Affiliations:** ^1^Department of Head and Neck Surgical Oncology, University Medical Center Utrecht, Utrecht, Netherlands; ^2^Department of Oral and Maxillofacial Surgery, University Medical Center Groningen, University of Groningen, Groningen, Netherlands; ^3^Department of Pathology and Medical Biology, University Medical Center Groningen, University of Groningen, Groningen, Netherlands; ^4^Department of Radiology, Nuclear Medicine and Anatomy, Radboud University Medical Center, Nijmegen, Netherlands; ^5^Department of Oral and Maxillofacial Surgery, Amsterdam University Medical Center, Amsterdam, Netherlands; ^6^Oral Pathology, Academic Center for Dentistry (ACTA) Amsterdam, Amsterdam, Netherlands; ^7^Department of Pathology, Amsterdam University Medical Center, Amsterdam, Netherlands; ^8^Department of Nuclear Medicine, The Netherlands Cancer Institute, Amsterdam, Netherlands; ^9^Department of Radiology and Nuclear Medicine, University Medical Center Utrecht, Utrecht, Netherlands; ^10^Department of Head and Neck Surgery, The Netherlands Cancer Institute, Amsterdam, Netherlands; ^11^Department of Otolaryngology-Head and Neck Surgery, Amsterdam University Medical Center, Amsterdam, Netherlands; ^12^Department of Pathology, University Medical Center Utrecht, Utrecht, Netherlands; ^13^Department of Otolaryngology-Head and Neck Surgery, Radboud University Medical Center, Nijmegen, Netherlands

**Keywords:** mouth neoplasms, sentinel lymph node biopsy, neck dissection, lymphatic metastasis, contralateral, recurrence, survival

## Abstract

**Introduction:** Nowadays, two strategies are available for the management of the clinically negative neck in early-stage (cT1-2N0) oral squamous cell carcinoma (OSCC): elective neck dissection (END) and sentinel lymph node biopsy (SLNB). SLNB stages both the ipsilateral and the contralateral neck in early-stage OSCC patients, whereas the contralateral neck is generally not addressed by END in early-stage OSCC not involving the midline. This study compares both incidence and hazard of contralateral regional recurrences (CRR) in those patients who underwent END or SLNB.

**Materials and Methods:** A retrospective multicenter cohort study, including 816 lateralized or paramedian early-stage OSCC patients, staged by either unilateral or bilateral END (*n* = 365) or SLNB (*n* = 451).

**Results:** The overall rate of occult contralateral nodal metastasis was 3.7% (30/816); the incidence of CRR was 2.5% (20/816). Patients who underwent END developed CRR during follow-up more often than those who underwent SLNB (3.8 vs. 1.3%; *p* = 0.018). Moreover, END patients had a higher hazard for developing CRR than SLNB patients (HR = 2.585; *p* = 0.030). In addition, tumor depth of invasion was predictive for developing CRR (HR = 1.922; *p* = 0.009). Five-year disease-specific survival in patients with CRR was poor (42%) compared to patients in whom occult contralateral nodal metastases were detected by SLNB or bilateral END (88%), although not statistically different (*p* = 0.066).

**Conclusion:** Our data suggest that SLNB allows for better control of the contralateral clinically negative neck in patients with lateralized or paramedian early-stage OSCC, compared to END as performed in a clinical setting. The prognosis of those in whom occult contralateral nodal metastases are detected at an earlier stage may be favorable compared to those who eventually develop CRR, which highlights the importance of adequate staging of the contralateral clinically negative neck.

## Introduction

In patients with early-stage (cT1-2N0) oral squamous cell carcinoma (OSCC), occult metastases are present in 20–30% of patients with a clinically negative neck, despite advanced diagnostic imaging modalities (1–3).

As watchful-waiting in these patients has been associated with a poor prognosis, especially when compared to those in whom the clinically negative neck was electively treated (1), two strategies are available for management of the clinically negative neck in early-stage OSCC: elective neck dissection (END) and sentinel lymph node biopsy (SLNB) (3–6). Although END is considered the best approach by many (5), SLNB has proven to reliably stage the clinically negative neck in early-stage OSCC with a pooled sensitivity and negative predictive value of 87 and 94%, respectively (4, 7–9). While END has the benefit of being a single-stage procedure, without need for specific facilities (e.g., nuclear medicine, advanced histopathology), SLNB is less invasive for the 70–80% of patients without metastatic neck involvement and has overall lower morbidity rates, better quality of life, and lower health care costs compared to END (10–13).

Furthermore, SLNB allows assessment of individual lymphatic drainage patterns and is able to detect aberrant drainage patterns (14, 15). This feature is of particular benefit in OSCC, since even lateralized OSCC occasionally metastasizes to contralateral cervical lymph nodes [2.7% (95% CI 1.2–4.2%)] (8, 9, 14, 16–21). Studies reported contralateral or bilateral lymphatic drainage patterns in 13–23% of lateralized OSCC patients, as detected during the SLNB procedure (8, 9, 14, 22).

Thus, SLNB stages the contralateral clinically negative neck in (lateralized) early-stage OSCC patients as well, whereas the contralateral clinically negative neck is generally not addressed by END in early-stage OSCC not involving the midline (i.e., lateralized or paramedian tumors).

Although the reported incidence of contralateral lymph node metastases in these patients is relatively low, underdiagnosis of the contralateral clinically negative neck is undesirable, especially since the presence of contralateral lymph node metastasis from OSCC has been associated with poor disease-specific survival (DSS) (16, 23, 24).

Therefore, this study aimed to assess whether SLNB allows for better control of the contralateral neck as compared to END, in early-stage OSCC not involving the midline. Accordingly, this study compares both incidence and hazard of contralateral regional recurrences (CRR) in those who underwent either END or SLNB as performed in daily clinical practice. Furthermore, this study compares the prognosis of those in whom occult contralateral nodal metastases were detected at an earlier stage by SLNB or bilateral END (pN2c) and those who eventually developed CRR.

## Materials and Methods

### Ethical Considerations

This study abided the Declaration of Helsinki and was approved by UMC Utrecht's Ethics Committee (no. 17/766) and all participating centers. The Internal Review Board waived requirement for signed informed consent forms for all subjects (4). Samples and data were handled according to General Data Protection Regulation.

### Patients

Patients without a history of head and neck cancer requiring treatment of the neck (i.e., neck dissection, neck irradiation) were included from five Dutch Head and Neck Cancer centers. In these centers, SLNB is currently part of standard oncological care in regard to staging the clinically negative neck in early-stage OSCC patients. Data were extracted from two large retrospective cohorts (END cohort and SLNB cohort), which have been extensively described by den Toom et al. (4).

For this study, only patients with early-stage OSCC (cT1-2N0) not involving the midline (i.e., lateralized or paramedian) were included in this study (AJCC UICC TNM-staging 7th Edition). Paramedian tumors were classified as tumors located adjacent to, but not involving, the midline. In all patients, clinical nodal staging was confirmed by palpation, imaging (i.e., ultrasound, CT, and/or MRI), and, in case of suspected lymph nodes, ultrasound-guided fine-needle aspiration cytology.

Patients who underwent unilateral END for tumors from which the specific location was missing were included. In these cases, it was estimated that performing unilateral END, instead of bilateral END, was on the basis of non-involvement of the midline. Patients who underwent bilateral END for confirmed lateralized or paramedian early-stage OSCC were included as well.

Patients were excluded if they underwent bilateral END for tumors from which the specific location was missing, as there was insufficient data to reliably assess whether the tumor involved the midline.

**Figure 1:**
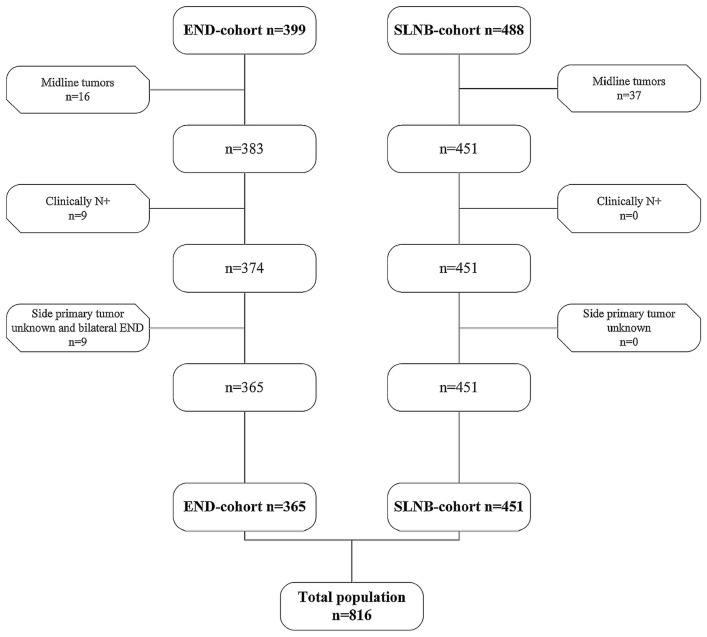
Flowchart for inclusion of patients in both the END cohort (*n* = 365) and the SLNB cohort (*n* = 451).

Out of 887 patients (END *n* = 399, SLNB *n* = 488), 816 patients met the inclusion criteria (END *n* = 365, SLNB *n* = 451) (Figure 1).

### Elective Neck Dissection

The END cohort has been previously described by den Toom et al. (4); early-stage OSCC patients who underwent END between 1990 and 2015 were included in the END cohort. END was performed as selective (level I–III/IV; *n* = 294) or modified radical neck dissection (level I–V; *n* = 70). Twenty-eight patients (7.7%) underwent bilateral END for lateralized or paramedian early-stage OSCC. The decision to perform either unilateral or bilateral END was made by the treating physician. The indication for bilateral END was on discretion of the treating physician and multidisciplinary team. END was elected over watchful-waiting when tumor depth of invasion (DOI) was estimated to be >4 mm (25). Neck dissection specimens were histopathologically assessed using conventional hematoxylin–eosin staining on formalin-fixed paraffin-embedded tissue.

### Sentinel Lymph Node Biopsy

Early-stage OSCC patients who underwent SLNB between 2007 and 2018 were included in the SLNB cohort. SLNB was performed according to European Association of Nuclear Medicine and Sentinel European Node Trial joint practice guidelines (26–28). SLNB was elected over watchful-waiting irrespective of tumor DOI. In short, the SLNB procedure consisted of preoperative peritumoral injections with technetium-99m [^99m^Tc]-labeled nanocolloid (80–240 MBq), followed by planar dynamic and static lymphoscintigraphy including SPECT-CT imaging, in a 1- or 2-day protocol. Intraoperative localization and extirpation of SLNs were performed using a handheld gammaprobe. Harvested SLNs were histopathologically assessed using step-serial sectioning (section thickness 150–500 μm) with hematoxylin–eosin staining and immunohistochemistry (26, 29). In SLNB-negative patients, a wait-and-scan policy was adopted, while SLNB-positive patients underwent complementary neck treatment. The vast majority of SLNB-positive patients underwent neck dissection as complementary neck treatment (85.6%; 89/104). Seven patients (6.7%) underwent complementary neck irradiation and three patients (2.9%) underwent complementary chemoradiation due to irradical resection of the primary tumor (*n* = 2) or presence of extracapsular spread of nodal metastasis (*n* = 1). Radiotherapy was employed only on the affected nodal basin in three patients, whereas in the other seven patients, the side and levels involved in neck irradiation were unknown.

### CRR, pN2c and Occult Contralateral Nodal Metastasis

Regional recurrences that occurred in the contralateral neck of the initial primary tumor, within 5 years following treatment, were regarded as event for CRR analyses. In addition, CRR in the presence of ipsilateral regional recurrences were regarded as event for CRR analyses as well. Regional recurrences in the presence of local recurrence or second primary tumors were excluded from final analyses, as differentiation between missed nodal metastasis at initial diagnostic work-up and metastasis developed from reseeding local recurrence is unfeasible.

Nodal metastasis detected in the contralateral neck of the primary tumor at time of initial neck staging, by either SLNB or bilateral END, was classified as pN2c, irrespective of the nodal status of the ipsilateral neck.

Occult contralateral nodal metastasis was defined as lymph node metastasis in the contralateral neck of the initial primary tumor, which was detected by either SLNB or bilateral END (i.e., pN2c) or which became clinically manifest during follow-up (i.e., CRR).

### Statistical Analysis

All data were analyzed with IBM SPSS Statistics Version 26.0. Data are expressed as mean ± SD for continuous variables. Number of cases and percentages are presented for categorical variables.

Independent samples *t* test was applied for parametric continuous variables, Mann–Whitney *U* test was applied for non-parametric continuous variables, and χ^2^ test was applied for categorical variables. Fisher's exact test was used to compare categorical variables containing small number of cases (*n* ≤ 5). *Post-hoc* testing was conducted in case of statistically significant χ^2^ test or Fisher's exact test outcomes for categorical variables with ≥3 groups.

For comparing 5-year DSS between patients with occult contralateral nodal metastasis (i.e., pN2c, CRR) and those without, Log-Rank test was conducted and Kaplan–Meier survival curves were computed. Furthermore, 5-year DSS were compared between patients in whom contralateral nodal metastases were detected by SLNB or bilateral END (pN2c) and those who eventually developed CRR during follow-up.

To assess independent predictors of CRR over time, Cox-regression analysis was applied (Method: Backward Likelihood Ratio). Variables that showed univariate association with occult contralateral nodal metastasis (i.e., pN2c and/or CRR), at a level of *p* ≤ 0.05, were included in the proportional hazard regression model. Accordingly, covariates were neck management (SLNB/END), initial ipsilateral pN+-status, location of primary tumor (i.e., paramedian or lateralized), vaso-invasive tumor growth, perineural tumor growth, and tumor DOI. Included covariates were analyzed for multicollinearity; variables with correlation of ≥0.5 were not included in Cox-regression analysis (30).

Missing data were handled by pairwise deletion. A *p*-value of <0.05 was regarded statistically significant.

## Results

The SLNB cohort contained a higher rate of tongue tumors (*p* < 0.001), whereas the END cohort contained a higher rate of floor-of-mouth tumors (*p* = 0.008) (Table 1). The END cohort had a higher rate of cT2-staged tumors (*p* < 0.001) and a higher rate of tumors staged pT2 or higher (52.8 vs. 24.6%; *p* < 0.001). Tumor DOI was higher in the END cohort (*p* < 0.001). Extracapsular spread of nodal metastases was more often present in the END cohort (*p* < 0.001). Median follow-up was longer for the END cohort (*p* < 0.001).

**Table 1:** Patient and tumor characteristics comparing END and SLNB cohort.

***N* = 806**	**SLNB (*n* = 451)**	**END (*n* = 365)**	*P*-value^*^
Age; mean (±SD)	62.03 (±11.97)	61.98 (±12.77)	0.960
Gender			0.533
Male (%)	233 (51.8%)	197 (54.0%)	
Site of primary tumor^a^			** <0.001**^†^**; 0.003**^†^
Tongue (%)	300 (66.5%)	195 (53.4%)	
Floor of Mouth (%)	98 (21.7%)	113 (31.0%)	
Buccal Mucosa (%)	34 (7.5%)	35 (9.6%)	
Other (%)	19 (4.3%)	22 (6.0%)	
cT-stage			** <0.001**^†^
T1 (%)	306 (67.8%)	133 (36.4%)	
T2 (%)	145 (32.2%)	222 (63.6%)	
pT-stage^b^			** <0.001**^†^
T1 (%)	340 (75.4%)	172 (47.2%)	
T2 (%)	107 (23.7%)	188 (51.5%)	
T3 (%)	4 (0.9%)	3 (0.8%)	
T4 (%)	0 (0%)	2 (0.5%)	
DOI; mean (±SD) in mm	5.32 (±4.28)	6.90 (±4.19)	** <0.001^‡^**
pN-stage			0.533
pN0 (%)	347 (76.9%)	274 (75.1%)	
pN+ (%)	104 (23.1%)	91 (24.9%)	
pN2c			0.199
Yes (%)	8 (1.8%)	2 (0.5%)	
ECS			** <0.001**[Table-fn TN4]
Yes (%)	3 (0.7%)	32 (8.8%)	
Follow-up in years; median (IQR)	2.2 (1.0–4.1)	4.6 (2.5–7.3)	** <0.001^˝^**

*SLNB sentinel lymph node biopsy, END elective neck dissection, SD standard deviation, DOI depth of invasion, ECS extracapsular spread, IQR interquartile range*.

* *Bold script indicates significant value*.

† *χ^2^ test*.

‡ *Independent samples t test*.

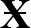
*Fisher's exact test*.

˝ *Mann-Whitney U test*.

a *Significance regarding tumors of the tongue and floor-of-mouth tumors*.

b *Significance regarding tumors staged pT2 or higher*.

### Contralateral Regional Recurrences

The overall rate of CRR was 2.5% (20/816). Tumor DOI was higher in patients who developed CRR (*p* < 0.001) (Table 2). Vaso-invasive tumor growth was more frequently present in patients who developed CRR (*p* = 0.032). END patients developed CRR more often (14/365; 3.8%) as compared to SLNB patients (6/451; 1.3%) (*p* = 0.021). None of the patients who underwent bilateral END developed CRR. In one patient, CRR was diagnosed in the presence of distant metastasis. CRR was diagnosed in the presence of ipsilateral regional recurrence in one END patient and in two SLNB patients. The rate of ipsilateral nodal metastases, as detected by END or SLNB, was higher in those who developed CRR (*p* = 0.018). None of the patients in whom occult contralateral nodal metastases were detected by SLNB or bilateral END (i.e., pN2c) developed CRR. Out of those who developed CRR, 15 patients underwent salvage treatment with curative intent; in three patients, no data on salvage treatment was available.

**Table 2:** Characteristics associated with contralateral regional recurrence.

***N* = 816**	**No CRR (*n* = 796)**	**CRR (*n* = 20)**	*P*-value^*^
Site of primary tumor			0.655
Tongue (%)	481 (60.4%)	14 (70.0%)	
Floor of Mouth (%)	206 (25.9%)	5 (25.0%)	
Buccal Mucosa (%)	68 (8.5%)	1 (5.0%)	
Other (%)	41 (5.2%)	0 (0%)	
pT-stage^a^			0.097
T1 (%)	503 (63.2%)	9 (45.0%)	
T2 (%)	286 (35.9%)	9 (45.0%)	
T3 (%)	5 (0.6%)	2 (10.0%)	
T4 (%)	2 (0.3%)	0 (0%)	
Location primary tumor			0.154
Lateralized	655 (97.4%)	18 (2.6%)	
Paramedian	23 (92.0%)	2 (8.0%)	
DOI; mean (±SD) in mm	5.90 (±4.21)	9.48 (±6.11)	** <0.001^‡^**
Non-cohesive growth			0.316
Yes (%)	267 (53.6%)	13 (65.0%)	
Perineural growth			0.071
Yes (%)	110 (18.8%)	7 (35.0%)	
Vasoinvasive growth			**0.032[Table-fn TN11]**
Yes (%)	51 (8.9%)	5 (25.0%)	
Procedure neck^b^			**0.021**^†^
SLNB (%)	445 (98.7%)	6 (1.3%)	
Unilateral END (%)	323 (95.8%)	14 (4.2%)	
Bilateral END (%)	28 (100%)	0 (0%)	
pN-stage			**0.018**^†^
Ipsilateral pN+ (%)	179 (22.5%)	9 (45.0%)	
pN2c			N.A.
Yes (%)	10 (1.3%)	0 (0%)	
ECS			0.588
Yes (%)	34 (4.3%)	1 (5.0%)	

*CRR contralateral regional recurrence, DOI depth of invasion, SD standard deviation, SLNB sentinel lymph node biopsy, END elective neck dissection, ECS extracapsular spread, N.A. not applicable*.

* *Bold script indicates significant value*.

† *χ^2^ test*.

‡ *Independent samples t test*.

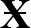
*Fisher's exact test*.

a *p value regarding tumors staged pT1 vs. pT2 or higher*.

b *Significance regarding difference in CRR rate between END and SLNB cohort*.

### Occult Contralateral Nodal Metastasis (i.e., pN2c and CRR)

The overall rate of occult contralateral nodal metastasis was 3.7% (30/816). Patients with paramedian tumors showed a higher rate of contralateral nodal metastases compared to those with lateralized tumors (*p* = 0.018) (Table 3). Tumor DOI was higher in patients with occult contralateral nodal metastasis (*p* < 0.002). Perineural tumor growth and vasoinvasive tumor growth were more often present in those with occult contralateral nodal metastasis (*p* = 0.002, *p* = 0.001). A higher rate of ipsilateral nodal metastases, as detected by SLNB or END, was seen in patients with occult contralateral nodal metastasis (*p* = 0.025). Of those in whom occult contralateral nodal metastasis was detected by either bilateral END or SLNB (i.e., pN2c), ipsilateral nodal metastasis was simultaneously detected in three patients (30%). No significant difference was seen in the rate of occult contralateral nodal metastasis between the END and SLNB cohort.

**Table 3:** Characteristics associated with occult contralateral nodal metastasis (i.e., pN2c and CRR).

***N* = 816**	**No contralateral metastases (*n* = 786)**	**Contralateral metastases (*n* = 30)**	*P*-value^*^
Site of primary tumor			0.394
Tongue (%)	474 (60.3%)	21 (70.0%)	
Floor of mouth (%)	203 (25.8%)	8 (26.7%)	
Buccal mucosa (%)	68 (8.7%)	1 (3.3%)	
Other (%)	41 (5.2%)	0 (0%)	
pT-stage^a^			0.277
T1 (%)	496 (63.1%)	16 (53.3%)	
T2 (%)	283 (36.0%)	12 (40.0%)	
T3 (%)	5 (0.6%)	2 (6.7%)	
T4 (%)	2 (0.3%)	0 (0%)	
Location primary tumor			**0.018[Table-fn TN15]**
Lateralized	657 (96.2%)	26 (3.8%)	
Paramedian	21 (84.0%)	4 (16.0%)	
DOI; mean (±SD) in mm	5.90 (±4.21)	8.46 (±5.75)	**0.002^‡^**
Non-cohesive growth			0.177
Yes (%)	262 (53.4%)	18 (66.7%)	
Perineural growth			**0.002**^^†^^
Yes (%)	106 (18.3%)	11 (42.3%)	
Vasoinvasive growth			**0.001**^^†^^
Yes (%)	49 (8.6%)	7 (28.0%)	
Procedure neck			0.334
SLNB (%)	437 (98.7%)	14 (3.1%)	
END (%)	349 (95.6%)	16 (4.4%)	
pN-stage^a^			**0.025**^^†^^
Ipsilateral pN+ (%)	176 (22.4%)	12 (40.0%)	
ECS			0.133
Yes (%)	32 (4.1%)	3 (10.0%)	

*CRR contralateral regional recurrence, DOI depth of invasion, SD standard deviation, SLNB sentinel lymph node biopsy, END elective neck dissection, ECS extracapsular spread*.

* *Bold script indicates significant value*.

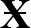
*Fisher's exact test*.

‡ *Independent samples t test*.

† *χ^2^ test*.

a *p-value regarding tumors staged pT1 vs. pT2 or higher*.

### Survival

**Figure 2:**
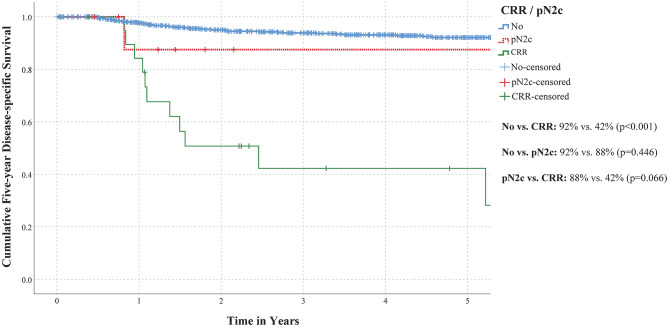
Five-year DSS curves for lateralized or paramedian early-stage OSCC patients without contralateral occult nodal metastasis (*blue bold line*) as compared to those with contralateral occult nodal metastasis: initially staged pN2c by SLNB or bilateral END (*red interrupted line*) or CRR (*green line*).

Figure 2 shows 5-year DSS for patients with and without occult contralateral nodal metastasis (i.e., pN2c and CRR). Five-year DSS was significantly shorter for patients who developed CRR as compared to patients without occult contralateral nodal metastasis (42 vs. 92%, *p* < 0.001). No difference in 5-year DSS was observed between those in whom occult contralateral nodal metastasis were detected by SLNB or bilateral END (i.e., pN2c) and patients without occult contralateral nodal metastasis (88 vs. 92%; *p* = 0.446). Five-year DSS of patients who developed CRR was worse compared to those in whom occult contralateral metastasis were detected by SLNB or bilateral END (i.e., pN2c), although not statistically significant (42 vs. 88%; *p* = 0.066). Of those who underwent salvage treatment with curative intent for CRR, 67% (10/15) died of disease after an average follow-up of 6.1 months following occurrence of CRR.

Mean time of survival in patients who developed CRR was 4.1 years (95% CI 2.29–5.95), whereas mean time of survival of those in whom contralateral nodal metastases were detected by SLNB or bilateral END (i.e., pN2c) was 9.7 years (95% CI 7.37–12.02). The mean time of survival in patients without occult contralateral nodal metastasis was 19.3 years (95% CI 18.81–19.72).

### Hazard for Developing CRR

**Figure 3:**
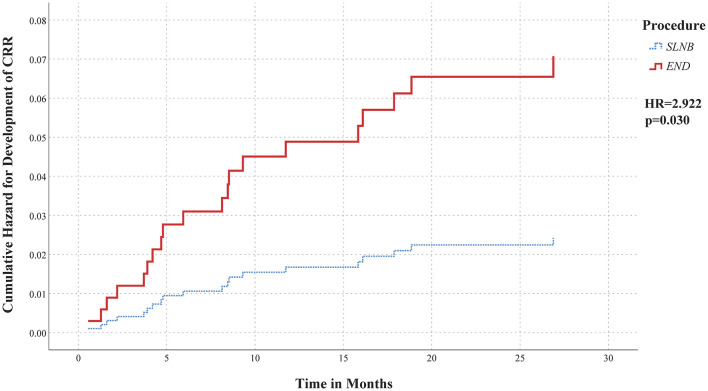
Cumulative hazard curve regarding development of CRR in patients with early-stage OSCC not involving the midline, divided by initial management of the neck: elective neck dissection (*END; red bold line*) or sentinel lymph node biopsy (*SLNB; blue interrupted line*). A significantly higher hazard for developing CRR was observed for patients who underwent END compared to those who underwent SLNB [HR = 2.922 (95% CI 1.11–7.71)].

Proportional hazard regression analysis showed that patients who underwent END had a higher hazard for developing CRR as compared to those who underwent SLNB [HR = 2.922 (95% CI 1.11–7.71); *p* = 0.030] (Figure 3). In addition, tumor DOI was significantly associated with development of CRR as well [HR = 2.277 (95% CI 1.44–3.60); *p* < 0.001].

## Discussion

This is the first study that evaluated incidence and hazard of CRR in early-stage OSCC not involving the midline (i.e., lateralized and paramedian) and compared these outcomes between patients who underwent either END or SLNB.

The overall incidence of occult contralateral nodal metastasis in this study was 3.7% (30/816), which is in concordance with the reported incidence of occult contralateral nodal metastasis in lateralized early-stage OSCC [2.7% (95% CI 1.2–4.2%)] (8, 9, 14, 16–21).

Our results showed higher incidence of CRR in patients who underwent END (3.8%) as compared to those who underwent SLNB (1.3%) (*p* = 0.018). Furthermore, our data showed that patients staged by END had a higher hazard of developing CRR, independent of factors such as tumor DOI, compared to patients staged by SLNB [HR = 2.922 (95% CI 1.11–7.71); *p* = 0.030].

Five-year DSS of patients who developed CRR was poor in our population, in particular when compared to those without occult contralateral nodal metastasis. These findings are in line with previous reports on prognosis of (lateralized) OSCC patients with CRR (16, 23, 24). Moreover, our results suggest that 5-year DSS of patients in whom contralateral nodal metastases were detected at an earlier stage by SLNB or bilateral END (pN2c) may be better than in those who eventually developed CRR. In addition, the successful salvage rate of those who developed CRR was only 33% in our population. This highlights the importance of adequate staging or treatment of the contralateral clinically negative neck.

Nevertheless, elective treatment of the contralateral clinically negative neck in OSCC without midline involvement remains controversial. This controversy is sustained by the varying incidence of occult contralateral nodal metastasis and CRR among institutions and the accompanying morbidity of (bilateral) END (18–20, 23, 24, 31–34). In our population, only two patients who underwent bilateral END had occult contralateral nodal metastasis, indicating that 26/28 patients (93%) underwent unnecessary contralateral END. With this in mind, it is worth noting that SLNB has the benefit of staging the contralateral clinically negative neck simultaneous with the ipsilateral neck. Accordingly, SLNB is able to avoid overtreatment of the contralateral neck by allowing accurate selection of only those that require treatment of the contralateral neck.

Another predictor for development of CRR in our population was tumor DOI [HR = 2.277 (95% CI 1.44–3.60); *p* < 0.001], which is in agreement with previous findings by Ganly et al. (35). In their study, neck failure in the undissected contralateral neck of T1-2N0 oral tongue patients accounted for 39% of all recurrences. Moreover, their results showed that tumor thickness was predicting for CRR. Although tumor thickness and DOI are not equivalent, they have similar prognostic implications for nodal metastases (36). As a consequence, the higher rate of CRR in our END cohort may be explained by greater tumor DOI in these patients. Nevertheless, when correcting for DOI in our proportional hazard regression analysis, a significantly higher hazard for developing CRR was observed in END patients as compared to SLNB patients.

The limitations of our study remain its retrospective design and the heterogeneity in performing SLNB or END among institutions. Secondly, occult contralateral nodal metastases are uncommon in this population, which irrevocably results in a small number of events for analyses. Accordingly, it could be argued that a larger sample, resulting in more CRR and pN2c events for analyses, may result in a significantly better prognosis for those in whom the metastatic involved contralateral neck is correctly staged and treated at an earlier stage, as compared to those who eventually develop CRR. Thirdly, since END patients were included between 1990 and 2015, a substantial proportion may have been elected for END based on potentially dated therapeutic guidelines or aged diagnostic imaging modalities. Moreover, patients were predominantly selected for END based on estimated tumor DOI >4 mm, inevitably resulting in higher tumor DOI in the END cohort. Due to this heterogeneity in therapeutic decision making between both cohorts, they cannot easily be compared, especially since the END cohort had a higher tumor DOI, higher T-stages, a higher rate of extracapsular spread of nodal metastases, and a longer follow-up duration, which might impact the occurrence of occult contralateral nodal metastasis or CRR. Nevertheless, there was no significant difference in the total rate of occult contralateral nodal metastasis (i.e., pN2c and CRR) between both cohorts, which implies that these cohorts can be compared when concerning control of the contralateral clinically negative neck. Furthermore, our proportional hazard regression analysis, which allows adjustment for confounding effects of included variables, showed a higher hazard for developing CRR in the END cohort, independent of confounding factors such as tumor DOI. In addition, both higher T-stages and presence of extracapsular spread of nodal metastases showed no association with contralateral nodal metastases or CRR in our univariate analyses. Besides, although a longer follow-up was available for END patient compared to SLNB patients, local or regional recurrences are uncommon after 2 years post-treatment (37). The follow-up duration of the SLNB cohort was therefore considered long enough for missed occult metastases to become clinically manifest and provides no explanation for the difference in rate of CRR between both cohorts. It could be argued that patients who underwent unilateral END for tumors from which the specific location was missing should be excluded from this study. However, since none of these patients developed CRR, excluding them would result in a relatively higher incidence of CRR in the END cohort, which will presumably induce a distortion of results in favor of SLNB. Fourthly, as there are no clear guidelines in which cases to perform contralateral END in early-stage OSCC, these were likely performed based on preference of the treating physician and on availability of the latest state-of-the-art imaging modalities. This may introduce some bias; however, it reflects daily clinical practice at that time. This strengthens the need for more research to develop evidence-based guidelines on this important topic. Fifthly, in this study, the 7th TNM classification was applied, whereas the 8th edition has already been implemented (38). While tumor diameter reflected pT-stage in the 7th edition, DOI is newly incorporated for T-stage in the 8th edition (36, 39). Due to missing data on DOI in several cases, our results could not be directly translated to the 8th TNM classification. Finally, some clinical and histopathological factors that have been associated with contralateral nodal metastasis in OSCC were not included due to lack of data. These factors include histological grading, surgical margin status, peritumoral inflammation, (adjuvant) radiotherapy to contralateral neck, and time of initial diagnosis (24). In particular, (adjuvant) radiotherapy to contralateral neck could influence the occurrence of CRR in these patients and should therefore be documented and incorporated in further studies. Although non-cohesive growth of the tumor was included as a potential predictor for CRR in our analyses, it was not subdivided by grading of pattern of invasion (i.e., cohesive growth, small islands, thin strands, and individual tumor cells) (24, 40). Nevertheless, the correlation between several of these factors (i.e., histological grading, peritumoral inflammation, and pattern of invasion) and contralateral nodal metastasis is dubious (24).

In conclusion, the incidence of CRR in lateralized or paramedian early-stage OSCC is relatively low (2.5%). As the salvage rate and prognosis of those who develop CRR remain poor, adequate staging of the contralateral clinically negative neck is highly recommended, especially since the prognosis of those in whom occult contralateral nodal metastases are detected at an earlier stage may be favorable compared to those who eventually develop CRR. In our population, a higher incidence of CRR was observed in those who underwent END for lateralized or paramedian early-stage OSCC, as compared to those who underwent SLNB. Furthermore, a higher hazard for developing CRR was observed in patients who underwent END in a clinical setting as compared to patients who underwent SLNB. Accordingly, our data suggest that SLNB allows for better control of the contralateral clinically negative neck in early-stage OSCC not involving the midline.

## Data Availability Statement

The raw data supporting the conclusions of this article will be made available by the authors, without undue reservation.

## Ethics Statement

The studies involving human participants were reviewed and approved by UMC Utrecht's Ethics Committee (no. 17/766) and all participating centers. Written informed consent for participation was not required for this study in accordance with the national legislation and the institutional requirements.

## Author Contributions

RM, IT, KB, RT, MW, and RB: conceptualization. RM, IT, KB, DL, and WK: methodology. RM, IT, and KB: software, formal analysis, and visualization. RM, IT, KB, and DL: validation. RM, IT, KB, DL, WK, MW, and RB: investigation. EB, MD, BK, WK, CL, SW, RT, MW, and RB: resources. IT, KB, DL, and WK: data curation. RM, IT, KB, MW, and RB: writing—original draft preparation. DL, EB, MD, BK, WK, CL, SW, and RT: writing—review and editing. MW and RB: supervision and project administration. IT and RB: funding acquisition. All authors have read and agreed to the published version of the manuscript.

## Conflict of Interest

The authors declare that the research was conducted in the absence of any commercial or financial relationships that could be construed as a potential conflict of interest.
